# Demonstrating the use of population level data to investigate trends in the rate, radiation dose and cost of Computed Tomography across clinical groups: Are there any areas of concern?

**DOI:** 10.1002/jmrs.811

**Published:** 2024-07-09

**Authors:** Sviatlana Kamarova, David Youens, Ninh T. Ha, Max Bulsara, Jenny Doust, Richard Fox, Marlene Kritz, Donald McRobbie, Peter O'Leary, Paul M. Parizel, John Slavotinek, Cameron Wright, Rachael Moorin

**Affiliations:** ^1^ Health Economics and Data Analytics, Curtin School of Population Health, Faculty of Health Sciences Curtin University Bentley Western Australia Australia; ^2^ Sydney School of Health Sciences The University of Sydney Sydney New South Wales Australia; ^3^ Nepean Blue Mountains Local Health District, New South Wales Health Kingswood New South Wales Australia; ^4^ Cardiovascular Epidemiology Research Centre, School of Population and Global Health The University of Western Australia Perth Western Australia Australia; ^5^ Institute for Health Research University of Notre Dame Notre Dame Western Australia Australia; ^6^ School of Population and Global Health The University of Western Australia Perth Western Australia Australia; ^7^ Australian Women and Girls' Health Research (AWaGHR) Centre, School of Public Health, Faculty of Medicine University of Queensland Brisbane Queensland Australia; ^8^ Division of Internal Medicine, Medical School, Faculty of Health and Medical Sciences The University of Western Australia Perth Western Australia Australia; ^9^ School of Physical Sciences University of Adelaide Adelaide South Australia Australia; ^10^ Obstetrics and Gynaecology Medical School, Faculty of Health and Medical Sciences The University of Western Australia Perth Western Australia Australia; ^11^ PathWest Laboratory Medicine QE2 Medical Centre Nedlands Western Australia Australia; ^12^ Medical School University of Western Australia Perth Western Australia Australia; ^13^ Department of Radiology Royal Perth Hospital Perth Western Australia Australia; ^14^ SA Medical Imaging, SA Health and College of Medicine and Public Health Flinders University Adelaide South Australia Australia; ^15^ Fiona Stanley Hospital Murdoch Western Australia Australia; ^16^ School of Medicine, College of Health and Medicine University of Tasmania Hobart Tasmania Australia

**Keywords:** Computed tomography, trends, utilisation

## Abstract

**Introduction:**

Increases in computed tomography (CT) use may not always reflect clinical need or improve outcomes. This study aimed to demonstrate how population level data can be used to identify variations in care between patient groups, by analysing system‐level changes in CT use around the diagnosis of new conditions.

**Methods:**

Retrospective repeated cross‐sectional observational study using West Australian linked administrative records, including 504,723 adults diagnosed with different conditions in 2006, 2012 and 2015. For 90 days pre/post diagnosis, CT use (any and 2+ scans), effective dose (mSv), lifetime attributable risk (LAR) of cancer incidence and mortality from CT, and costs were assessed.

**Results:**

CT use increased from 209.4 per 1000 new diagnoses in 2006 to 258.0 in 2015; increases were observed for all conditions except neoplasms. Healthcare system costs increased for all conditions but neoplasms and mental disorders. Effective dose increased substantially for respiratory (+2.5 mSv, +23.1%, *P* < 0.001) and circulatory conditions (+2.1 mSv, +15.4%, *P* < 0.001). The LAR of cancer incidence and mortality from CT increased for endocrine (incidence +23.4%, mortality +18.0%) and respiratory disorders (+21.7%, +23.3%). Mortality LAR increased for circulatory (+12.1%) and nervous system (+11.0%) disorders. The LAR of cancer incidence and mortality reduced for musculoskeletal system disorders, despite an increase in repeated CT in this group.

**Conclusions:**

Use and costs increased for most conditions except neoplasms and mental and behavioural disorders. More strategic CT use may have occurred in musculoskeletal conditions, while use and radiation burden increased for respiratory, circulatory and nervous system conditions. Using this high‐level approach we flag areas requiring deeper investigation into appropriateness and value of care.

## Graphical abstract

This repeated cross‐sectional study examined trends in CT use, costs and associated radiation dosages between high‐level clinical groups. CT use increased for most patient groups though the degree of increase varied between groups, while trends in costs and radiation dosages varied substantially between clinical groups. This work highlights areas which may require further research to determine the appropriateness and value of care being delivered.

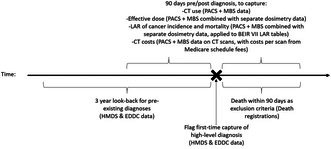

## Introduction

Computed tomography (CT) is instrumental for diagnosis and clinical management of numerous conditions. CT use has been increasing rapidly over recent decades across many countries including the USA and Australia.^1^, ^2^, ^3^, ^4^ However, many studies indicate that this increase does not correspond with improvements in diagnostic yield or growth in patient volume.^1^, ^2^, ^5^, ^6^ This may indicate low value care,^7^ that is, some CT use may be inappropriate and potentially cause unnecessary radiation exposure.^8^, ^9^

While individual risks of cancer incidence and mortality due to medical radiation are relatively small,^10^ increasing use may cause problems at the population level.^11^, ^12^ This issue has gained international attention, with calls for increased clinical awareness and practice change.^13^, ^14^ Cumulative radiation dose, which may be a particular concern for individuals having repeated CT scans, poses a significant risk of cancer,^14^, ^15^, ^16^ warranting dose monitoring and reduction strategies. Many studies have evaluated trends in CT use (i) in specific clinical conditions including injury,^17^ cancer,^18^ cardiac diseases and endovascular repair,^19^, ^20^ Crohn's^21^ and kidney disease;^22^ (ii) across anatomical areas (e.g. head^23^ and abdomen^24^); and (iii) across healthcare settings.^20^, ^25^, ^26^ One study has assessed variation in CT use across clinical conditions among British paediatrics,^27^ though there is a lack of evidence in the general population concerning variation in CT use and the associated burden in terms of costs, cumulative radiation dose and risk of cancer and mortality.

Therefore, this study aimed to demonstrate how population level data can be used to identify potential variations in care between high‐level patient groups, to flag areas requiring deeper investigation into appropriateness and value of care being delivered. This is demonstrated via an analysis of system‐level changes in the use of CT around the diagnosis of new conditions according to broad diagnostic chapters in terms of rate, cost, effective dose and lifetime attributable risk (LAR) of cancer incidence and mortality in Western Australia.

## Methods

This population‐based retrospective repeated cross‐sectional study follows the REporting of studies Conducted using Observational Routinely‐collected health Data (RECORD) statement.^28^ Approval was granted by the Curtin University Human Research Ethics Committee (HREC), Western Australia (WA) Department of Health HREC (2011/97) and the Australian Institute of Health and Welfare HREC (EO2018/4/485), under a waiver of consent.

### Data sources

This study is part of a larger project using person‐level linked administrative data including a cohort of all West Australians: (i) aged 18+ who had a separation (discharge) from any WA hospital for any non‐pregnancy‐related condition, or (ii) who presented to any public hospital emergency department (ED) for any reason, or (iii) who had a CT scan undertaken in WA by a public or private provider between 1 January 2003 and 1 December 2016. The study includes two types of data: person‐level de‐identified administrative linked data and data pertaining to the average radiation dose of the CT protocol used.

#### Administrative data sets

Using State‐based data from the WA Data Linkage System^29^ and Australian Medicare Benefits Schedule (MBS) data provided by the Australian Institute of Health and Welfare (AIHW), cross‐jurisdictional (i.e. State – Commonwealth) privacy‐preserving record linkage was undertaken by the Curtin University Centre for Data Linkage.^30^ The following data were available:

1. Hospital Morbidity Data Set (HMDS) records from 2003 to 2016 for all discharges from any WA hospital (public or private) for conditions excluding pregnancy, including dates of admission and discharge, hospital type (tertiary or secondary) and diagnosis (principal and co‐diagnoses).
2. Emergency Department Data Collection (EDDC) records 2003–2016 for all WA public hospital ED presentations, including presentation date, diagnosis code and major diagnostic group.
3. WA Picture archiving and communication system (PACS) data for all CT scans (excluding positron emission tomography/CT (PET/CT)) from January 2003 to May 2016 undertaken in all WA public tertiary hospitals and selected public secondary hospitals as an out‐patient, ED patient or admitted patient. Records included the scan date, and the type (including the protocol code) of CT examination undertaken (e.g. non‐contrast head CT, etc.). The Medicare Benefit Schedule (MBS) item code was available for CTs undertaken from 2013.
4. MBS data capturing all CT scans (excluding PET/CT) subsidised by the Federal Australian Government (i.e. performed outside of hospital or in hospital (public or private) for private patients), including the date and the type of scan and the MBS item number and fee (i.e. fee payable to providers for the examination by the Australian Commonwealth Government^31^), between 2003 and 2016.
5. WA Death Registration records from 2003 to 2016 including the age and date of death.

#### Dosimetry data set

Technical CT data were gathered for various CT protocols as part of a prior project.^32^ Under the previous project, technical data were obtained in 2011 for adult diagnostic CT scanning protocols undertaken at five public hospitals in WA,^32^ where a random sample of 20 cases of each protocol were sampled per hospital (i.e. a total of 100 scans per protocol). Twenty cases per protocol were used in this study as this is the standard practice for estimating typical doses delivered by scanning protocols, and exceeds European Guidelines on the collection of dosimetry data for development of dose reference levels for CT.^33^ In cases where a protocol was applied fewer than 20 times during the study period, all cases were selected to be included.^32^ Protocol information contained separate scanning sequences whenever present. Technical data parameters collected were kilovoltage (kV), milliamperage (mA), tube rotation times, collimation width, pitch, scanning method, anatomical reference start‐stop positions, volume weighted CT dose index (CTDIvol), dose‐length product (DLP) and scanner model. The average sex‐specific organ specific dose (milligrays, (mGy)) and effective dose (millisieverts (mSv)) for each protocol (cumulated from the sequences where required) were calculated using the National Cancer Institute dosimetry system for Computed Tomography (NCICT).^34^

**Figure 1:**
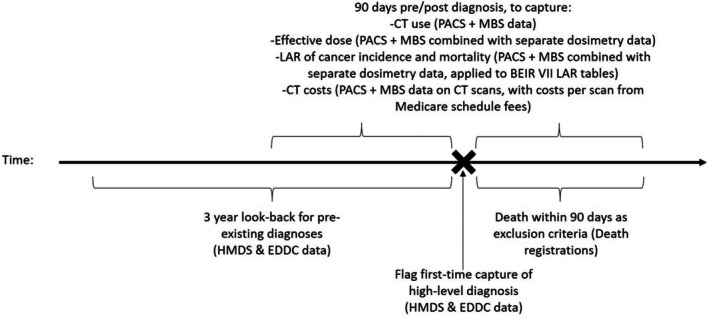
Study design presenting data sources and key pieces of information from each. Bottom part describes cohort selection criteria, top part describes outcomes captured for analysis. BEIR, Biologic Effects of Ionizing Radiation; CT, Computed Tomography; EDDC, Emergency Department Data Collection; HMDS, Hospital Morbidity Data Set; LAR, lifetime attributable risk; MBS, Medicare Benefits Schedule; PACS, Picture Archiving and Communications System.

Figure 1 notes the key pieces of information utilised from each data set to capture the cohort for analysis, and ascertain the outcome variables.

### Study population

The study population were people aged 18+ years who had an ED presentation or hospital admission in 2006, 2012 or 2015 with no hospitalisations/ED presentations in the prior three calendar years. We separately identified: (i) people with history of hospitalisations/ED presentations in the 3 years prior to each study year (due to complexity in assigning use of CT between different diagnoses), the ‘prevalent cohort’; and (ii) people with no hospitalisations/ED presentations in the year under examination (due to lack of diagnostic information from which to assign to CT use), the ‘no hospitalisation cohort’. Because this study focuses on the use of CT prior to and following the development of a new diagnosis, and these groups lack evidence of the development of new diagnoses, they were omitted from most analyses. We excluded individuals who died during hospitalisation or within 90 days following ED presentation/hospital discharge due to limited follow‐up.

### Categorisation of index events

The first‐time hospitalisation/ED presentation in each of the 3 years under examination was deemed the index event for that year. Index events were then classified into the following broad diagnostic chapters according to their principal diagnosis recorded in either the ED or HMDS data using the International Statistical Classification of Diseases, which is regarded as a useful classification structure for general epidemiological purposes.^35^ We use the following groups using the tenth revision,^35^ Australian Modification (ICD‐10‐AM) codes:

- Infectious and parasitic diseases (A, B);
- Neoplasms, in situ and unknown (C, D00‐49);
- Blood, blood forming and immune system (D50‐89);
- Endocrine, nutritional and metabolic system (E);
- Mental and behavioural disorders (F);
- Nervous system (G);
- Eye diseases (H00‐50);
- Ear diseases (H60‐95);
- Circulatory system (I);
- Respiratory system (J);
- Digestive system (K);
- Skin and subcutaneous tissue diseases (L);
- Musculoskeletal and connective tissue (M);
- Genitourinary system (N);
- Injury, poisoning and other external causes (S, T).

In those admitted to hospital directly from ED the diagnosis on the HMDS was used to classify the index event.

### CT use in diagnostic window

CT scans were captured from both the PACS and MBS data. An individual scan may appear in both data sets in cases where a private patient is treated in a public hospital or when a patient is referred by a general practitioner to a public hospital as an outpatient. Where a patient had multiple scans of the same protocol recorded on the same day, only one scan was counted. The number of CT scans were characterised through an ascertainment period of 90 days prior and 90 days following the index event date. The rate of any CT and repeated CT (2+) during the ascertainment period is reported as the number of CT scans per 1000 index events. To enable comparison of the prevalent/no hospitalisation cohorts compared to the study population (Table S1), CTs were also captured for the prevalent cohort (relative to the first hospitalisation in each study year) and the no hospitalisation cohort (using an index date randomly assigned in each study year).

### Cost of CT scanning

Costs were assessed as the cost to the government only (both state and federal). The cost of CT scanning within the ascertainment period for each index event was calculated from the MBS schedule fee applicable to the item number of the CT scan at the time of the scan, adjusted to 2021 Australian Dollars using the Consumer Price Index. This approach was used to apply costs for out‐of‐hospital scans and in‐hospital scans, in the absence of information on the actual costs of performing CT in hospital. At the time of writing, one Australian dollar was worth 0.59 Euros and 0.64 US dollars. Prior to 2013, where PACS data did not include equivalent MBS item numbers, the MBS item relevant to the same PACS code in post‐2013 data was applied. Where a pre‐2013 PACS record could not be mapped to a single MBS item, the mean schedule fee for all CT scans of the same anatomical area was applied.

### Cancer incidence and mortality

For each CT scan in the data set, the effective dose and organ‐specific radiation doses described earlier were applied based on scan type and patient sex. The sex and age specific LAR of cancer incidence and mortality resulting each CT scan was estimated using the protocol specific organ dose from the dosimetry data set and the age/sex‐specific risk coefficients from Tables 12D‐1 and 12D‐2 of the biological effects of ionising radiation (BEIR) VII report.^36^ Note that as incidence and mortality LAR estimates for the whole study period are based on dosimetry data fixed at 2012 values, these provide a surrogate for the potential health impacts of changes in utilisation (i.e. examination types, number of scans, patient age at time of scan), not actual population risk estimates. Note that LAR for incidence and mortality are calculated and reported separately. The LAR of cancer incidence and mortality resulting from radiation dose to the remainder and ‘other’ organs was calculated using doses for organs not named in the BEIR VII LAR tables but which have a weight by International Commission on Radiological Protection (ICRP) 103,^37^ or are included in the remainder organs by ICRP 103, and weighting them by the risk attributed by BEIR VII for ‘other’ organs. This method assumes all such organs contribute equally to risk. The analysis was repeated for CT scans undertaken on persons aged 18 to 80 years using linear interpolation of the BEIR VII risk coefficients from the two nearest tabulated ages when data were not available for a specific age. For persons aged over 80 years, the linear extrapolation for each protocol was extended until the estimated number of cancers or mortality reached zero.

### Covariates

Socio‐demographic characteristics included sex, age group at date of index event (18–44, 45–64, and 65+ years), and Indigenous status. Accessibility to services was measured using the Accessibility/Remoteness of Index of Australia (ARIA)^38^ based on the patient's postcode at the index date. Socio‐economic status was classified using the Socio‐Economic Indexes For Areas – Index of Relative Socio‐Economic Disadvantage (SEIFA‐IRSD) based on postcode, for the census closest to the index date, categorised as quintiles.^39^ Comorbidity was determined using the Multipurpose Australian Comorbidity Scoring System (MACSS^40^) capturing the number of 17 MACSS conditions recorded on any hospitalisation across the 5 years prior to the index date.

### Analysis

Descriptive statistics were used to compare characteristics of the study population to the prevalent and no hospitalisation cohorts. For the prevalent and no hospitalisation groups, we report available demographic characteristics and rates of CT use in comparison to the study population to better understand generalisability; remaining analyses were conducted in the study population only. For each diagnostic group, the following metrics were used to describe the burden of CT scanning within the diagnostic window of index events observed in 2006, 2012 and 2015: number (and percentage) of individuals having any and multiple (rate of CT use within individuals with 2+ CT scans), the rate of CT scanning per 1000 index events, average and total cost of CT scanning. Demographic characteristics within the study population were also reported at each time point to better gauge whether changes in the study population may have impacted CT use trends. Effective dose (in mSv) was calculated following summation within individuals of the effective doses of all CT scans within the ascertainment period. Both the average effective dose and the LAR of cancer incidence and mortality attributable to CT scanning undertaken within the ascertainment period were calculated by limiting to individuals recording at least one CT scan. For each year, the diagnostic groups were ranked according to the magnitude of each metric. Changes in the absolute value of each metric and changes in the ranking were calculated across the 3 years, with statistical significance of changes from 2006 to 2015 assessed by *t*‐tests and Wald tests.

## Results

A total of 504,723 adults were included. Approximately, half of the study population (50.6%) were male or in the 18–44 age group (46.9%). The majority of the population lived in major cities (73.8%) and approximately one‐third in areas of least disadvantage (29.3%) (Table 1). The distribution of characteristics was similar across the three study periods, except for a greater proportion living in areas of lowest socio‐economic disadvantage in 2012 relative to the other study years (*P* < 0.001), and a relatively lower proportion living in Major Cities in 2006 (*P* < 0.001). The study population was similar to the prevalent/no hospitalisation cohorts in most characteristics, except for age: the prevalent cohort were older and the no hospitalisation cohort were younger (*P* < 0.001, Table S1). Rates of CT use were also higher in the study population than the other cohorts (in 2015 258.01 per 1000 index event for study population, 234.46 per 1000 in prevalent cohort and 42.14 in no hospitalisation cohort, *P* < 0.001). Other characteristics also reported statistically significant differences across the cohorts; however, these differences were small (Table 1, Table S2). Across major diagnostic groups (Table 1), injury, poisoning and other external causes made‐up the highest percentage of index events (23.2%), followed by digestive system (16.7%), musculoskeletal and connective tissue (11.1%) and genitourinary system disorders (8.3%). Small but significant differences were observed over time; for example, injury, poisoning and other external causes increased from 22.6% in 2006 to 23.9, while digestive system disorders decreased slightly from 18.3% to 16.1%. There were some differences in clinical features between males and females, for example, males had more comorbidities (*P* < 0.001) and the diagnostic chapters differed (*P* < 0.001) though these did not change substantially within either sex over time (Table S2).

**Table 1:** Demographic characteristics and CT use among study population.

Characteristics	2006 (*n* = 142,875)		2012 (*n* = 180,311)		2015 (*n* = 182,663)		Total (*n* = 504,723)		*P*‐value^1^
	*n*	%	*n*	%	*n*	%	*n*	%	
Sex of patient									
Male	72,442	50.7	91,943	51.0	91,120	50.2	255,505	50.6	<0.001
Female	70,413	49.3	88,333	49.0	90,384	49.8	249,130	49.4	
Indeterminate/not stated	20	0.0	35	0.0	33	0.0	88	0.0	
Age group									
18–44 years	66,667	46.7	86,975	48.2	83,308	45.9	236,950	46.9	<0.001
45–64 years	50,232	35.2	61,337	34.0	62,393	34.4	173,962	34.5	
65+ years	25,976	18.2	31,999	17.8	35,836	19.7	93,811	18.6	
Diagnostic chapters									
Injury and poisoning	32,213	22.6	41,569	23.1	43,361	23.9	117,143	23.2	<0.001
Digestive system	26,082	18.3	29,207	16.2	29,176	16.1	84,465	16.7	
Musculoskeletal system	15,387	10.8	20,147	11.2	20,445	11.3	55,979	11.1	
Genitourinary system	12,481	8.7	14,246	7.9	15,312	8.4	42,039	8.3	
Neoplasms	9985	7.0	12,251	6.8	13,426	7.4	35,662	7.1	
Circulatory system	9791	6.9	11,304	6.3	9411	5.2	30,506	6.0	
Eye and adnexa	7080	5.0	9883	5.5	10,429	5.7	27,392	5.4	
Skin and connective tissues	6615	4.6	8940	5.0	7858	4.3	23,413	4.6	
Respiratory system	6129	4.3	8973	5.0	8262	4.6	23,364	4.6	
Nervous system	4094	2.9	5713	3.2	5960	3.3	15,767	3.1	
Ear and mastoid processes	3635	2.5	4944	2.7	3568	2.0	12,147	2.4	
Infectious and parasitic disease	3281	2.3	5715	3.2	5659	3.1	14,655	2.9	
Endocrine, nutritional and metabolic diseases	2708	1.9	3098	1.7	3642	2.0	9448	1.9	
Mental and behavioural disorders	2604	1.8	3429	1.9	4047	2.2	10,080	2.0	
Blood/ blood forming	790	0.6	892	0.5	981	0.5	2663	0.5	
Socio‐economic status^2^									
Lowest disadvantage	38,090	26.7	62,790	34.8	47,141	26.0	148,021	29.3	<0.001
Low disadvantage	23,748	16.6	27,738	15.4	35,779	19.7	87,265	17.3	
Moderate disadvantage	30,365	21.3	35,886	19.9	33,540	18.5	99,791	19.8	
High disadvantage	27,894	19.5	36,401	20.2	40,301	22.2	104,596	20.7	
Highest disadvantage	15,103	10.6	16,091	8.9	23,760	13.1	54,954	10.9	
Unknown	7675	5.4	1405	0.8	1016	0.6	10,096	2.0	
Accessibility to services^3^									
Major cities	99,080	69.4	133,513	74.1	139,879	77.1	372,472	73.8	<0.001
Inner regional	17,495	12.2	16,651	9.2	15,521	8.6	49,667	9.8	
Outer regional	14,771	10.3	15,219	8.4	13,875	7.6	43,865	8.7	
Remote	7917	5.5	10,548	5.9	7128	3.9	25,593	5.1	
Very remote	3394	2.4	3782	2.1	4260	2.4	11,436	2.3	
Unknown	218	0.2	598	0.3	874	0.5	1690	0.3	
Comorbidities^4^									
0–1	47,127	33.0	63,557	35.3	63,554	35.0	174,238	34.5	<0.001
2–5	91,335	63.9	111,291	61.7	111,537	61.4	314,163	62.2	
6+	4413	3.1	5463	3.0	6446	3.6	16,322	3.2	
Outcome variables									
Individuals with at least one CT	18,714	13.1	23,912	13.3	27,133	15.0	69,759	13.8	<0.001
Individuals with 2+ CT	6667	4.7	9037	5.0	11,148	6.1	26,852	5.3	<0.001
Rate of any CT use (per 1000 index events)	209.36		214.71		258.01		228.77		<0.001
Rate of CT use (per 1000 individuals with 2+ CT index events)	2679.62		2638.04		2767.58		2702.15		<0.001
Average individual cost (and SD) of CT scan per index event (in AUD^5^)	84.91	287.59	74.19	246.34	82.03	254.85	80.04	261.67	0.002
Total cost of CT scans during ascertainment period (sum, in millions AUD^5^)	12.13	n/a	13.38	n/a	14.89	n/a	40.40	n/a	n/a
Mean effective dose (and SD) in adults with at least one CT (mSv^6^)	13.6	14.8	13.4	15.1	14.2	16.1	13.7	15.4	<0.001
Cancer incidence for individuals with any CT (per 100,000 individuals)^7^	77.1	99.1	74.2	99.3	76.7	105.9	75.9	101.9	0.931
Cancer mortality for individuals with any CT (per 100,000 individuals)^7^	46.7	57.1	45.5	57.4	47.5	61.3	46.6	58.9	0.983

^1^ Displaying significance of changes in 2006 compared to 2015.

^2^ Socio‐economic status measured by SEIFA‐IRSD: Socio‐Economic Indexes for Areas, Index of Relative Socio‐Economic Disadvantage.

^3^ Accessibility to services was measured using ARIA: Accessibility and Remoteness Index of Australia.

^4^ Comorbidity: Number of Multipurpose Australian Comorbidity Scoring System conditions reported in hospitalisation data in the 5 years prior to the index event date.

^5^ AUD: Australian dollars adjusted to 2021 values using the Consumer Price Index.

^6^ Mean of effective dose accumulated within individuals across all scans during ascertainment period.

^7^ LAR: Lifetime risk of cancer attributable to CT scanning undertaken during ascertainment period.

The rate of any CT use and 2+ CT use increased from 2006 to 2015 by 48.6 and 87.9 per 1000 respectively (*P* < 0.001 for both; Table 1). Compared to the baseline, while mean costs associated with CT per individual decreased in 2015 (from $84.91 to $82.03, *P* = 0.002), the total cost of CT scanning for the cohort consistently increased over the same period ($AU 12.13 million in 2006 to 14.89 million in 2015). The cumulative effective dose per individual slightly decreased from 14 mSv in 2006 to 13 mSv in 2012, then increased to 14 mSv in 2015; the change from 2006 to 2015 reached statistical significance (*P* < 0.001). Similarly, the LAR of incidence and mortality of cancer initially decreased in 2012, then rebounded in 2015 to just under the baseline level by 0.4/100,000 individuals (incidence) or beyond the baseline by 0.8/100,000 (mortality), though the changes from 2006 to 2015 were not significant (Table 1). Differences by sex are presented in Table S2.

### Changes over time

#### CT use

Table 2 presents the ranking of diagnostic groups in terms of the rate of CT use and rate of CT use for individuals with 2+ CTs. The top three groups in 2006 were neoplasms, nervous system, and circulatory system for any CT use; these remained the top three in 2015 though in reverse order. A statistically significant 2.0% decrease in CT rate was recorded from 2006 to 2015 for neoplasms, while no significant change was recorded for blood and blood forming organs, musculoskeletal system, mental and behavioural disorders or infectious and parasitic diseases. The remaining 10 diagnostic groups recorded significant increases, the largest in disorders of the ear and mastoid process (+109.1%, *P* < 0.001) and disorders of the eye and adnexa (+61.8%, *P* < 0.001).

**Table 2:** Change in rates of any CT and 2+ CT use from 2006 to 2015.

Rate of any CT use									
Ranking			Major clinical chapters	Rate of CT use^1^			Change in 2015 vs. 2006		
2006	2012	2015		2006	2012	2015	Absolute value	%	Significance level
1	2	3	Neoplasms	492.34	410.91	427.98	−66.36	−2.0	***
2	1	2	Nervous system	406.69	432.52	464.77	60.5	14.7	**
3	3	1	Circulatory system	349.81	369.16	548.29	198.16	56.0	***
4	5	4	Respiratory system	279.00	242.17	310.70	32.76	11.7	*
5	6	5	Blood and blood forming	245.57	233.18	291.54	46.73	19.1	
6	7	9	Musculoskeletal system	215.12	203.36	219.71	4.32	2.0	
7	8	8	Mental and behavioural disorders	214.29	190.43	221.40	14.47	5.8	
8	4	6	Genitourinary system	189.57	246.74	264.30	74.5	39.1	***
9	11	7	Injury and poisoning	173.44	186.77	246.40	73.79	42.5	***
10	10	11	Endocrine, nutritional and metabolic diseases	162.11	188.51	212.25	51.35	31.4	***
11	9	10	Digestive system	161.84	189.89	215.86	55.04	34.0	***
12	12	13	Infectious and parasitic disease	126.18	118.11	142.25	17.08	13.4	
13	13	12	Ear and mastoid process	99.04	114.28	206.56	108.06	109.1	***
14	14	14	Eye and adnexa	59.32	75.08	96.17	36.72	61.8	***
15	15	15	Skin and connective tissues	49.43	57.61	73.05	23.11	46.4	***

Rate of CT use among individuals with 2+ CTs									
2006	2012	2015	Major clinical chapters	2006	2012	2015	Absolute value	%	Significance level
1	2	1	Injury and poisoning	3032.85	2892.12	3232.83	199.98	6.6	***
2	1	2	Circulatory system	2896.67	2977.6	2991.62	94.95	3.3	***
3	3	3	Neoplasms	2858.66	2849.04	2979.66	121	4.2	***
4	7	8	Infectious and parasitic disease	2542.86	2434.43	2503.5	−39.36	−1.5	
5	5	5	Respiratory system	2518.62	2507.73	2619.97	101.35	4.0	***
6	4	13	Endocrine, nutritional, and metabolic diseases	2500	2530.77	2394.59	−105.41	−4.2	***
7	6	4	Nervous system	2482.47	2502.58	2676.29	193.82	7.8	***
8	8	11	Digestive system	2455.36	2425.75	2442.08	−13.28	−0.5	
9	9	9	Musculoskeletal system	2467.49	2420.29	2501.71	34.22	1.4	***
10	11	6	Blood and blood forming	2413.04	2326.09	2610.17	197.13	8.2	***
11	10	14	Eye and adnexa	2323.08	2410.85	2329.61	6.53	0.3	
12	15	15	Genitourinary system	2298.08	2320.83	2325.09	27.01	1.2	**
13	12	7	Ear and mastoid processes	2285.71	2364.34	2526.88	241.17	10.6	***
14	13	12	Mental and behavioural disorders	2236.64	2434.78	2418.7	182.06	8.1	***
15	14	10	Skin and connective tissues	2291.67	2388.89	2491.23	199.56	8.7	***

Ranking is highest (1) to lowest (15) based on magnitude of the metric (i.e. rate).

^1^ All rates are per 1000 index events, *P < 0.05, **P < 0.01, ***P < 0.001, estimated using *t*‐test.

In terms of CT use among those with 2+ CTs, the groups' injury and poisoning, circulatory system and neoplasms had the highest rates in 2006 and 2015. An increase in CT use among those with 2+ CTs was observed in neoplasms despite an overall reduction in CT use in this group. Overall, CT use among individuals with 2+ CTs increased, except for a reduction for endocrine, nutritional and metabolic diseases (−4.2%, *P* < 0.001, in contrast to an increase in any CT use in this group), and no change in infectious and parasitic diseases, digestive system disorders, and disorders of the eye and adnexa.

#### Costs

Total costs of CT significantly increased during the study period for all groups but neoplasms and mental disorders, which had non‐significant changes (Table 3). Neoplasms had the highest costs in all years (mean $2.5 million). The largest increases were observed for eye (104.8%), infectious (68.6%), endocrine (52.8%) and ear (55.3%) disorders. The largest absolute increase occurred for injury ($0.678 million increase). The top ranked conditions changed with injury moving up to second position and circulatory system ranked fourth in 2015, after increasing by 40.6% and 35.0%, respectively.

**Table 3:** Cost^1^ and mean effective dose^2^ associated with CT scanning during ascertainment period, 2006 to 2015.

Ranking			Major clinical chapters	Cost of CT scanning^1^			Change in 2015 vs. 2006		
2006	2012	2015		2006	2012	2015	Absolute value	%	Significance level
1	1	1	Neoplasms	2.598	2.400	2.518	−0.080	−3.1	
2	2	3	Digestive system	2.055	2.183	2.294	0.239	11.6	***
3	3	2	Injury and poisoning	1.671	1.924	2.349	0.678	40.6	***
4	5	5	Musculoskeletal system	1.29	1.437	1.504	0.214	16.6	***
5	4	4	Circulatory system	1.267	1.458	1.710	0.443	35.0	***
6	6	6	Genitourinary system	0.998	1.238	1.365	0.367	36.8	***
7	7	7	Respiratory system	0.719	0.786	0.879	0.160	22.3	***
8	8	8	Nervous system	0.469	0.579	0.634	0.165	35.2	***
9	9	10	Infectious and parasitic disease	0.191	0.288	0.322	0.131	68.6	***
10	11	11	Endocrine, nutritional and metabolic disease	0.178	0.226	0.272	0.094	52.8	***
11	10	9	Eye and adnexa	0.166	0.257	0.340	0.174	104.8	***
12	14	14	Mental and behavioural disorders	0.163	0.155	0.189	0.026	16.0	
13	12	12	Skin and connective tissues	0.145	0.190	0.199	0.054	37.2	***
14	13	13	Ear and mastoid processes	0.123	0.159	0.191	0.068	55.3	***
15	15	15	Blood and blood forming	0.097	0.099	0.127	0.030	30.9	*

Ranking			Major clinical chapters	Mean effective dose^2^			Change in 2015 vs. 2006		
2006	2012	2015		2006	2012	2015	Absolute value	%	Significance level
1	1	1	Neoplasms	22.3	22.6	22.6	0.3	1.4	
2	2	2	Blood and blood forming	18.3	19.6	20.1	1.8	10.1	
3	3	3	Digestive system	16.1	16.2	16.8	0.6	3.9	
4	6	5	Genitourinary system	13.7	13.2	14.0	0.3	2.0	
5	4	4	Circulatory system	13.5	14.5	15.6	2.1	15.4	***
6	5	6	Endocrine, nutritional and metabolic diseases	12.0	13.4	13.4	1.4	11.6	
7	7	7	Infectious and parasitic disease	11.9	13.2	13.2	1.3	10.6	
8	9	10	Skin and connective tissues	11.5	11.1	11.4	−0.1	−0.7	
9	10	9	Injury and poisoning	11.4	10.7	12.6	1.2	10.3	*
10	11	11	Musculoskeletal system	10.6	10.4	10.5	−0.1	−1.2	
11	8	8	Respiratory system	10.6	11.3	13.1	2.5	23.1	***
12	12	12	Eye and adnexa	8.5	8.6	9.8	1.3	15.7	*
13	13	13	Nervous system	7.3	7.7	8.5	1.2	17.0	**
14	14	15	Mental and behavioural disorders	6.0	6.5	6.1	0.1	1.3	
15	15	14	Ear and mastoid process	5.9	5.6	6.9	1.0	16.6	

Ranking is highest (1) to lowest (15) based on magnitude of the metric (i.e. total cost, mSv).

^1^ Costs presented in millions of Australian dollars adjusted to 2021 values using the Consumer Price Index, *P < 0.05, **P < 0.01, ***P < 0.001, estimated using Wald test.

^2^ Mean of individual effective dose (in mSv) accumulated within individuals across all scans during ascertainment period, *P < 0.05, **P < 0.01, ***P < 0.001, estimated using *t*‐test.

#### Radiation exposure

There was an overall increase in the mean of effective dose accumulated across all scans in the ascertainment period; however, there were only five groups where this change was statistically significant. Statistically significant increases were recorded for respiratory conditions (+2.5 mSv, +23.1%, *P* < 0.001), circulatory conditions (+2.1 mSv, +15.4%, *P* < 0.001), eye and adnexa (+1.3 mSv, +15.7%, *P* = 0.03), nervous system disorders (+1.2 mSv, +17.0%, *P* = 0.003) and injury and poisoning (+1.2 mSv, +10.3%, *P* < 0.05). There was no change over time in the top‐ranking positions (Table 3).

#### Change in LAR attributable to CT use

LAR of cancer incidence/mortality attributable to CT scanning demonstrated minor changes in ranking positions. The incidence of cancer attributable to CT significantly reduced among musculoskeletal conditions from 2006 to 2015 (−10.0%, *P* < 0.01). The largest statistically significant increases in both LARs were observed for endocrine (incidence +23.4%, *P* = 0.01; mortality +18.0%, *P* = 0.04) and respiratory disorders (incidence +21.7%, mortality +23.3%, both *P* < 0.001). There was also a significant increase in risks of mortality among circulatory (*P* = 0.01) and nervous system diseases (*P* = 0.048) (Table 4).

**Table 4:** Lifetime risk of cancer attributable to CT during ascertainment period, change from 2006 to 2015.

Incidence (among cohort members with any CT)									
Ranking			Major clinical chapters	LAR^1^			Change in 2015 vs. 2006		
2006	2012	2015		2006	2012	2015	Absolute value	%	Significance level
1	1	1	Neoplasms	117.6	118	116.7	−0.9	−0.8	
2	3	3	Digestive system	92.1	90.4	93.1	1	1.1	
3	2	2	Blood and blood forming	89.1	92.3	94.1	5	5.6	
4	4	4	Genitourinary system	85.1	80.5	83.7	−1.4	−1.6	
5	6	5	Injury and poisoning	76.6	68.3	77	0.4	0.5	
6	5	6	Infectious and parasitic disease	72.8	80.4	75.7	2.9	4.0	
7	9	10	Skin and connective tissues	66.8	61.7	63	−3.8	−5.7	
8	10	11	Musculoskeletal system	63.8	59.5	57.4	−6.4	−10.0	**
9	8	8	Circulatory system	62.6	65.1	66.9	4.3	6.9	
10	7	7	Endocrine, nutritional and metabolic diseases	60.6	66.5	74.8	14.2	23.4	*
11	11	9	Respiratory system	52.9	55.1	64.4	11.5	21.7	***
12	12	12	Nervous system	45.2	47	48.1	2.9	6.4	
13	13	15	Mental and behavioural disorders	37.9	40.6	35.9	−2	−5.3	
14	14	13	Eye and adnexa	37.5	36.0	39.6	2.1	5.6	
15	15	14	Ear and mastoid processes	37.6	33.6	39.5	1.9	5.1	

Mortality (among cohort members with any CT)									
2006	2012	2015	Major clinical chapters	2006	2012	2015	Absolute value	%	Significance level
1	1	1	Neoplasms	75.7	76.7	76.1	0.4	0.5	
2	2	2	Blood and blood forming	56.6	60.9	62.4	5.8	10.2	
3	3	3	Digestive system	54.6	54	55.8	1.2	2.2	
4	5	4	Genitourinary system	48.7	46.3	48.6	−0.1	−0.2	
5	4	7	Infectious and parasitic disease	43.6	47.4	45.7	2.1	4.8	
6	8	9	Injury and poisoning	43	38.9	44.4	1.4	3.3	
7	6	5	Circulatory system	42.8	45.1	48	5.2	12.1	*
8	10	10	Skin and connective tissues	39.7	37.5	39	−0.7	−1.8	
9	7	6	Endocrine, nutritional and metabolic diseases	39.4	43.6	46.5	7.1	18.0	*
10	9	8	Respiratory system	36.1	37.7	44.5	8.4	23.3	***
11	11	11	Musculoskeletal system	35.7	34.3	33.8	−1.9	−5.3	
12	12	12	Nervous system	27.3	29.1	30.3	3	11.0	*
13	14	13	Eye and adnexa	24.9	24.2	27.1	2.2	8.8	
14	13	15	Mental and behavioural disorders	22.4	24.3	21.3	−1.1	−4.9	
15	15	14	Ear and mastoid processes	22.2	20.6	24.9	2.7	12.2	

Ranking is highest (1) to lowest (15) based on magnitude of the metric (incidence and mortality rate).

Incidence: estimated LAR^1^ of any cancer type, per 100,000 individuals, *P < 0.05, **P < 0.01, ***P < 0.001.

Mortality: estimated number of deaths due to cancer of any type^1^, per 100,000 individuals, *P < 0.05, **P < 0.01, ***P < 0.001, using *t*‐test.

## Discussion

Australia, having the second‐highest rate of CT use of Organisation for Economic Co‐operation and Development countries in 2017–2021,^41^ provides a useful setting to examine CT trends. We found that CT use and costs increased across the majority of diagnostic chapters, as with previous studies in WA.^1^, ^2^, ^42^, ^43^ Use of CT should be guided by the ‘as low as reasonably achievable’ (ALARA) principle for radiation protection,^44^ according to which the likelihood of exposure and number of exposed persons should be as low as reasonably achievable. If the increases in use reported here are driven by factors other than clinical need, this would represent a violation of this principle. With this high‐level analysis, we therefore identify areas where further research may be required to assess the appropriateness and value of care. We also provide some discussion of factors that may contribute to these findings, which may also help to guide further research.

Although provider‐level factors can drive CT use, such as fear of missed diagnoses,^45^ there are also some system‐level factors in Australia. One is the high number of CT scanners in the country.^41^ Additionally, Australia has greater restrictions on use of magnetic resonance imaging (MRI) than CT, for example, restrictions on general practitioner referral for MRI.^46^ In 2012, a ‘Four‐hour rule’ was introduced, under which ED attendances should be resolved within 4 hours;^47^ this may promote CT for rapid diagnosis and hence admission or discharge. Additionally, an activity‐based funding (ABF) model was introduced in August 2011 in place of block payments, which may contribute to our findings,^1^, ^42^, ^48^ as ABF can promote inappropriate early discharge and hence readmission with further diagnostic testing.^49^

We observed some demographic changes in the study population from 2006 to 2015, which may partially explain some results. The most notable was a higher proportion living in major cities in 2015 compared to 2006. The Diagnostic Imaging Review Reform Package, implemented in Australia from 2011 to 2016, had a focus on improving availability of imaging for those in rural and remote areas on account of lower rates of use in this population^50^; hence, the increase in the proportion living in major cities may have made a small contribution to overall increases, while for those remaining in rural areas access also improved. Other demographic factors recorded only minor changes despite being statistically significant; the significant findings may reflect the large size of the study population rather than clinically important population changes.

Consistent with existing evidence,^14^, ^15^, ^16^, ^51^ we found that the increase in CT from 2006 to 2015 coincided with significantly increased radiation burden for some conditions (respiratory system, circulatory system, injury and poisoning, nervous system and eye and adnexa disorders). Since our study used fixed doses at the protocol level derived from 2011 data, changes in radiation dose cannot be due to changes in dose delivery (i.e. low‐dose techniques) or improved dose optimisation. The only mechanisms that can affect doses reported here are (i) changes in the number of CT examinations and (ii) changes in CT examination types.

In general, increases in the rate of CT among those with 2+ CTs were smaller than the increases in rates of any CT, and for two conditions (neoplasms and endocrine, nutritional and metabolic disorders) these rates moved in opposite directions. This suggests that changes in costs, effective dose and cancer risks may have been generally driven by an increase in the population having any CT rather than an increase in repeated CT. Previous research has indicated that repeated CT is more likely in distinct subgroups of patients,^52^ which may explain why trends in the 2+ CT group did not closely reflect overall trends.

Neoplasms ranked highest for both cumulative effective dose and associated cancer risks across the study years. However, this was the only chapter to record a reduction in use in 2015 compared to baseline and moved from the first to the third position in CT use. Similarly, this was one of two chapters without increasing costs. Studies prior to and during the timeframe of the current study have cautioned against excessive CT in some cancer patients. For example, Harvey et al. reported minimal benefit of thoracic CT in surveillance of stage I testicular cancer due to its limited benefit,^53^ while Lecouvert et al. reported that for patients with high‐risk prostate cancer, whole body MRI performed as well as CT for evaluating lymph node metastases, highlighting the potential radiation risks that may be averted by use of MRI instead of CT.^54^ More recently, Choosing Wisely Australia has advised against tests for recurrent cancer in treated, asymptomatic patients in the absence of evidence for benefit.^55^ These trends may also be driven by an increasing use of PET/CT in cancer management.^56^ As our available data did not include PET/CT scans, it is possible that the reduction in CT use among those with cancer observed reflects a substitution by PET/CT. Considering that cancer survival continues to improve,^57^, ^58^ clinical practices and policies need to minimise radiation exposure to prevent development of secondary cancers linked to medical imaging.

Use of CT in musculoskeletal conditions significantly increased for those having 2+ CT scans between 2006 and 2015, although the associated risk of cancer incidence significantly declined; this significant risk reduction was not observed for any other chapter. This may indicate more appropriate use of diagnostic imaging for musculoskeletal disorders.^42^ It is likely that these trends reflect practice change within this clinical chapter, attenuating increases in individual cumulated effective doses. Strategies such as more selective practice (e.g. multiple/repeat scanning for anatomic structures that are further away from the torso but careful consideration of CT use of the axial and appendicular skeleton associated with substantially elevated radiation exposures^59^, ^60^ or substitution to MRI^61^, ^62^) may explain findings.

We observed a concerning pattern of increased health risks attributable to upsurges and/or changes in utilisation of CT within respiratory, nervous and circulatory diagnostic conditions, where individual effective dose and LAR of cancer mortality increased significantly. Furthermore, individuals diagnosed with injuries or eye disorders recorded an increasing effective dose, without significant changes in LAR, while endocrine disorder patients had increasing risks of incidence and mortality attributed to CT with no change in effective dose. Further research may be required to determine whether these trends have continued, and if so, what may be driving increases in radiation in these areas. Assessment of whether these trends relate to specific subgroups may be informative, for example if use in these clinical areas was found to have increased among younger patients this may explain the increased LAR while also highlighting areas for potential intervention. Similarly, research into scanning practice, for example, adherence to clinical decision rules where these exist for these conditions, would highlight possible interventions to reduce patient risks involved with CT use.

### Strengths and limitations

Since dosimetry data were collected in 2011, analyses do not account for changes in radiation dosages within protocols over time. This would be a limitation if the study was designed to determine the impact of technological (or practice) change on doses received from CT. However, since we aimed to determine how changes in CT utilisation affect effective dose and cancer risk, using a standardised protocol‐based dose estimate means that any differences observed are due to utilisation rather than CT technique. Given the study aim, this is a benefit rather than limitation. Evaluation of changes in radiation dose according to CT study type over time would be a priority for future study in areas identified here as reporting substantial increases in utilisation and hence dose – since dose reduction techniques may have counteracted the purely volume driven dose estimates we have reported. Dosimetry information is not currently collected within the PACS; the collection of such data could facilitate important future research into CT‐associated radiation risks.

We cannot assess if observed trends are justified. However, high‐level work of this nature can uncover trends that require further investigation to improve care. This can be observed in work undertaken in the area of unwarranted variation, where atlases of variation have been instrumental in promoting evidence‐based care, reducing unnecessary care, and improving safety, efficiency and outcomes.^63^, ^64^ The study period ends in 2015. As imaging data are not routinely linked in Western Australia a lengthy approvals process was required to facilitate an ad hoc, cross‐jurisdictional linkage. Given the hypothesis‐generating nature of this work, which aims to support further research, the age of data may be less of a concern. As our cohort selection was based on first‐time ED presentation or hospital admission for a given condition, findings are not generalisable to the population managed entirely in the community where hospital or ED use does not occur. Findings are also not generalisable to those with prior hospital/ED use (i.e. prevalent individuals); we did observe that the study population differed from the prevalent and no hospitalisation cohorts in some demographic characteristics and had higher rates of CT use. Our analysis of costs uses Medicare costs so captures costs to the government only. About 10% of the costs of medical imaging in Australia are patient out‐of‐pocket costs.^65^ Any CT scans which are unnecessary therefore risk placing an additional financial burden on patients.

## Conclusion

Trends in CT use, radiation burden and health risks varied across diagnostic chapters, though the overall increase aligns with policy‐driven explanations proposed in earlier literature. Use declined for neoplasms, possibly due to practice change driven by radiation concerns among cancer patients. Concerning practices of increasing CT use and radiation burden occurred in respiratory, circulatory and nervous system conditions. Costs also increased for all chapters, except for neoplasms and mental and behavioural disorders. Future research should investigate whether these trends have continued, and the factors potentially driving these trends, in particular in those clinical areas where we identify increases in radiation risks through the study period.

### Funding Statement

This study was funded with an Australian National Health and Medical Research Council grant, project grant APP1144573. The study funders had no role in the study design, conduct, manuscript writing or decision to submit for publication.

## Author Contributions

SK, DY, NTH, MB, JD, JS, DM, MK, POL, RF, PP and RM contributed to the study design and concept. RM contributed to the acquisition of data. RM, SK, DY, MB, JD, JS, DM, POL, CW, DM2, TC, PP, RF and NTH contributed to the analysis and interpretation of the data. SK, RM and DY contributed to drafting of the manuscript. RM, JD, JS, DM, POL, DY, CW, DM2, PP, RF and NTH contributed to critical revision of the manuscript for important intellectual content. RM, SK, DY and MK contributed to statistical expertise. RM, MB, JD, DM, POL, JS and RF secured funding for the study.

## Conflict of Interest

The authors have no conflicts of interest to declare. The institutions of RM, NH, POL, DY, CW, MB, RF and DM received grant funding from the National Medical Research Council of Australia for investigator‐initiated research. The funding agreement ensured author independence in designing the study, interpreting the data, writing and publishing the report.

## Ethical Approval and Patient Consent Statement

Ethics approval was provided by the Curtin University Human Research Ethics Committee (HRE2017‐0822), the Australian Institute of Health and Welfare Human Research Ethics Committee (EO2018/4/485), and the Western Australian Department of Health Human Research Ethics Committee (2011/97). This project was approved under a waiver of consent.

## Supporting information


**Table S1.** Descriptive characteristics of the index hospitalisation, prevalent, no hospitalisation cohorts in 2006, 2012.
**Table S2.** Demographic characteristics and CT use of the study population according to gender.

## Data Availability

The data that support the findings of this study are available from the relevant data custodians of the study data sets. Restrictions by the data custodians mean that the data are not publicly available or able to be provided by the authors. Researchers wishing to access the data sets used in this study should refer to the Western Australian Data Linkage Unit and the Australian Institute of Health and Welfare.
