# Supplementary material for: Demonstrating the use of population level data to investigate trends in the rate, radiation dose and cost of Computed Tomography across clinical groups: Are there any areas of concern?
Source: J Med Radiat Sci. 2024 Jul 9;72(Suppl 2):S16–30. doi: 10.1002/jmrs.811 (PMC12449605; doi:10.1002/jmrs.811)
Supplement: Supplementary file 1 — Table S1. Descriptive characteristics of the index hospitalisation, prevalent, no hospitalisation cohorts in 2006, 2012. Table S2. Demographic characteristics and CT use of the study population according to gender. [file JMRS-72-S16-s001.docx]

**Supporting information** *Demonstrating the use of population level data to investigate trends in the rate, radiation dose and cost of CT scanning across clinical groups: Are there any areas of concern?*

**Table S1. Descriptive characteristics of the index hospitalisation, prevalent, no hospitalisation cohorts in 2006, 2012 and 2015**

|  | **Study population** | | | | | | **Prevalent cohort** | | | | | | **No hospitalisation cohort^3^** | | | | | |  |
| --- | --- | --- | --- | --- | --- | --- | --- | --- | --- | --- | --- | --- | --- | --- | --- | --- | --- | --- | --- |
| **Characteristics** | **2006**  **(n = 142875)** | | **2012**  **(n = 180311)** | | **2015**  **(n = 181537)** | | **2006**  **(n = 207369)** | | **2012**  **(n = 286920)** | | **2015**  **(n = 303930)** | | **2006**  **(n = 1474961)** | | **2012**  **(n = 1466084)** | | **2015**  **(n = 1452248)** | | **Sig.^4^** |
|  | **n** | **%** | **n** | **%** | **n** | **%** | **n** | **%** | **n** | **%** | **n** | **%** | **n** | **%** | **n** | **%** | **n** | **%** |  |
| ***Sex of patient*** |  |  |  |  |  |  |  |  |  |  |  |  |  |  |  |  |  |  |  |
| Male | 72442 | 50.7 | 91943 | 51.0 | 91120 | 50.2 | 103817 | 50.1 | 143712 | 50.1 | 150753 | 49.6 | 724825 | 49.2 | 716449 | 48.9 | 711145 | 49.0 | p<0.001 |
| Female | 70413 | 49.3 | 88333 | 49.0 | 90384 | 49.8 | 103544 | 49.9 | 143200 | 49.9 | 153169 | 50.4 | 749845 | 50.8 | 749353 | 51.1 | 740824 | 51.1 |  |
| Indeterminate/ not stated | 20 | 0.0 | 35 | 0.0 | 33 | 0.0 | 8 | 0.0 | 8 | 0.0 | 8 | 0.0 | 180 | 0.0 | 171 | 0.0 | 167 | 0.0 |  |
| ***Age groups*** |  |  |  |  |  |  |  |  |  |  |  |  |  |  |  |  |  |  |  |
| 18-44 years | 66667 | 46.7 | 86975 | 48.2 | 83308 | 45.9 | 76307 | 36.8 | 104639 | 36.5 | 107102 | 35.2 | 890304 | 60.4 | 813916 | 55.5 | 750659 | 51.7 | p<0.001 |
| 45-64 years | 50232 | 35.2 | 61337 | 34.0 | 62393 | 34.4 | 65960 | 31.8 | 91877 | 32.0 | 94952 | 31.2 | 421632 | 28.6 | 452971 | 30.9 | 472996 | 32.6 |  |
| 65+ years | 25976 | 18.2 | 31999 | 17.8 | 35836 | 19.7 | 65102 | 31.4 | 90404 | 31.5 | 101876 | 33.5 | 163025 | 11.1 | 199197 | 13.6 | 228593 | 15.7 |  |
| ***Socio-economic status***^1^ |  |  |  |  |  |  |  |  |  |  |  |  |  |  |  |  |  |  |  |
| Lowest disadvantage | 38090 | 26.7 | 62790 | 34.8 | 47141 | 26.0 | 44542 | 21.5 | 81096 | 28.3 | 65585 | 21.6 | 455638 | 30.9 | 476386 | 32.5 | 429331 | 29.6 | p<0.001 |
| Low disadvantage | 23748 | 16.6 | 27738 | 15.4 | 35779 | 19.7 | 30165 | 14.6 | 40154 | 14.0 | 52898 | 17.4 | 254721 | 17.3 | 242635 | 16.6 | 259019 | 17.8 |  |
| Moderate disadvantage | 30365 | 21.3 | 35886 | 19.9 | 33540 | 18.5 | 44456 | 21.4 | 56493 | 19.7 | 56028 | 18.4 | 284145 | 19.3 | 280163 | 19.1 | 270980 | 18.7 |  |
| High disadvantage | 27894 | 19.5 | 36401 | 20.2 | 40301 | 22.2 | 48961 | 23.6 | 71445 | 24.9 | 71918 | 23.7 | 264317 | 17.9 | 284936 | 19.4 | 290864 | 21.0 |  |
| Highest disadvantage | 15103 | 10.6 | 16091 | 8.9 | 23760 | 13.1 | 31735 | 15.3 | 36118 | 12.6 | 56264 | 18.5 | 168245 | 11.4 | 145993 | 10.0 | 168133 | 11.6 |  |
| Unknown | 7675 | 5.38 | 1405 | 0.8 | 1016 | 0.6 | 7510 | 3.6 | 1614 | 0.6 | 1237 | 0.4 | 47784 | 3.2 | 35860 | 2.5 | 33809 | 2.3 |  |
| ***Accessibility to services***^2^ |  |  |  |  |  |  |  |  |  |  |  |  |  |  |  |  |  |  |  |
| Major cities | 99080 | 69.4 | 133513 | 74.1 | 139879 | 77.1 | 123124 | 59.4 | 184380 | 64.3 | 202988 | 66.8 | 1044199 | 70.8 | 1077012 | 73.5 | 1065263 | 73.4 | p<0.001 |
| Inner regional | 17495 | 12.2 | 16651 | 9.2 | 15521 | 8.6 | 25862 | 12.5 | 32470 | 11.3 | 31217 | 10.3 | 171679 | 11.7 | 143577 | 9.8 | 140071 | 9.7 |  |
| Outer regional | 14771 | 10.3 | 15219 | 8.4 | 13875 | 7.6 | 30650 | 14.8 | 36090 | 12.6 | 35674 | 11.7 | 122404 | 8.3 | 114011 | 7.8 | 114159 | 7.9 |  |
| Remote | 7917 | 5.5 | 10548 | 5.9 | 7128 | 3.9 | 17742 | 8.6 | 22854 | 8.0 | 18765 | 6.2 | 79408 | 5.4 | 74539 | 5.8 | 71970 | 5.0 |  |
| Very remote | 3394 | 2.4 | 3782 | 2.1 | 4260 | 2.4 | 9745 | 4.7 | 10629 | 3.7 | 14163 | 4.7 | 36739 | 2.5 | 35879 | 2.5 | 37012 | 2.6 |  |
| Unknown | 218 | 0.2 | 598 | 0.3 | 874 | 0.5 | 246 | 0.1 | 497 | 0.2 | 1123 | 0.4 | 20421 | 1.4 | 20955 | 1.4 | 23661 | 1.6 |  |
| ***Outcome variables*** |  |  |  |  |  |  |  |  |  |  |  |  |  |  |  |  |  |  |  |
| Rate of any CT use (per 1,000 index events) | 209.36 |  | 214.71 |  | 258.01 |  | 175.18 |  | 199.46 |  | 234.46 |  | 27.73 |  | 34.99 |  | 42.14 |  | p<0.001 |
| Rate of CT use (per 1,000 individuals with 2+ CT scans) | 2679.62 |  | 2638.04 |  | 2767.58 |  | 2560.67 |  | 2571.12 |  | 2625.45 |  | 2236.07 |  | 2266.91 |  | 2284.57 |  | p=0.002 |

*^1^ Socio-economic status measured by SEIFA-IRSD: Socio-Economic Indexes for Areas, Index of Relative Socio-Economic Disadvantage*

*^2^ Accessibility to services was measured using ARIA: Accessibility and Remoteness Index of Australia*

*^3^ For the cohort excluded due to no hospitalisations, an index date has been randomly selected within each study year to allow the calculation of CT use through the ascertainment period*

*^4^ p-values report significance of comparisons between groups in 2015*

**Table S2. Demographic characteristics and CT use of the study population according to gender**

|  | **Female** | |  |  |  |  |  |  |  | **Male** |  |  |  |  |  |  |  |  |  |
| --- | --- | --- | --- | --- | --- | --- | --- | --- | --- | --- | --- | --- | --- | --- | --- | --- | --- | --- | --- |
|  | **2006**  **(n = 70413)** | | **2012**  **(n = 88333)** | | **2015**  **(n = 90384)** | | **Total**  **(n = 249130)** | | **Sig.^7^** | **2006**  **(n = 72442)** | | **2012**  **(n = 91943)** | | **2015**  **(n = 91120)** | | **Total**  **(n = 255505)** | | **Sig.^7^** | **Sig.^8^** |
|  | **n** | **%** | **n** | **%** | **n** | **%** | **n** | **%** |  | **n** | **%** | **n** | **%** | **n** | **%** | **n** | **%** |  |  |
| ***Age groups*** |  |  |  |  |  |  |  |  |  |  |  |  |  |  |  |  |  |  |  |
| 18-44 years | 31838 | 45.2 | 41296 | 46.8 | 40518 | 44.8 | 113652 | 45.6 | p<0.001 | 34821 | 48.1 | 45667 | 49.7 | 42783 | 47.0 | 123271 | 48.2 | p<0..001 | p<0.001 |
| 45-64 years | 24573 | 34.9 | 29933 | 33.9 | 30957 | 34.3 | 85463 | 34.3 |  | 25649 | 35.4 | 31386 | 34.1 | 31418 | 34.5 | 88453 | 34.6 |  |  |
| 65+ years | 14002 | 19.9 | 17104 | 19.4 | 18909 | 20.9 | 50015 | 20.1 |  | 11972 | 16.5 | 14890 | 16.2 | 16919 | 18.6 | 43781 | 17.1 |  |  |
| ***Diagnostic chapter*** |  |  |  |  |  |  |  |  |  |  |  |  |  |  |  |  |  |  |  |
| Blood and blood forming | 545 | 0.8 | 619 | 0.7 | 682 | 0.8 | 1846 | 0.7 | p<0.001 | 245 | 0.3 | 273 | 0.3 | 299 | 0.3 | 817 | 0.3 | p<0.001 | p<0.001 |
| Circulatory system | 4149 | 5.9 | 4846 | 5.5 | 4061 | 4.5 | 13056 | 5.2 |  | 5641 | 7.8 | 6455 | 7.0 | 5346 | 5.9 | 17442 | 6.8 |  |  |
| Digestive system | 13460 | 19.1 | 14864 | 16.8 | 14704 | 16.3 | 43028 | 17.3 |  | 12621 | 17.4 | 14342 | 15.6 | 14465 | 15.9 | 41428 | 16.2 |  |  |
| Ear and mastoid processes | 1785 | 2.5 | 2340 | 2.7 | 1785 | 2.0 | 5910 | 2.4 |  | 1849 | 2.6 | 2603 | 2.8 | 1783 | 2.0 | 6235 | 2.4 |  |  |
| Endocrine, nutritional, and metabolic diseases | 1788 | 2.5 | 2188 | 2.5 | 2,280 | 2.9 | 6604 | 2.7 |  | 919 | 1.3 | 910 | 1.0 | 1014 | 1.1 | 2843 | 1.1 |  |  |
| Eye and adnexa | 3490 | 5.0 | 4901 | 5.6 | 5,403 | 6.0 | 13794 | 5.5 |  | 3589 | 4.9 | 4981 | 5.4 | 5026 | 5.5 | 13596 | 5.3 |  |  |
| Genitourinary system | 9317 | 13.2 | 10437 | 11.8 | 11,177 | 12.4 | 30931 | 12.4 |  | 3163 | 4.4 | 3807 | 4.1 | 4133 | 4.5 | 11103 | 4.3 |  |  |
| Infectious and parasitic disease | 1747 | 2.5 | 3168 | 3.6 | 3,282 | 3.6 | 8197 | 3.3 |  | 1534 | 2.1 | 2547 | 2.8 | 2377 | 2.6 | 6458 | 2.5 |  |  |
| Injury and poisoning | 12011 | 17.1 | 16270 | 18.4 | 17456 | 19.3 | 45737 | 18.4 |  | 20192 | 27.8 | 25276 | 27.5 | 25887 | 28.4 | 71355 | 27.9 |  |  |
| Mental and behavioural disorders | 1295 | 1.8 | 1742 | 2.0 | 1,933 | 2.1 | 4970 | 2.0 |  | 1309 | 1.8 | 1686 | 1.8 | 2114 | 2.3 | 5109 | 2.0 |  |  |
| Musculoskeletal system | 7052 | 10.0 | 9156 | 10.4 | 9,392 | 10.4 | 25600 | 10.3 |  | 8333 | 11.5 | 10989 | 12.0 | 11052 | 12.1 | 30374 | 11.9 |  |  |
| Neoplasms | 5503 | 7.8 | 6462 | 7.3 | 7,143 | 7.9 | 19108 | 7.7 |  | 4482 | 6.2 | 5789 | 6.3 | 6283 | 6.9 | 16554 | 6.5 |  |  |
| Nervous system | 2257 | 3.2 | 2893 | 3.3 | 2,973 | 3.3 | 8123 | 3.3 |  | 1837 | 2.5 | 2820 | 3.1 | 2986 | 3.3 | 7643 | 3.0 |  |  |
| Respiratory system | 3043 | 4.3 | 4556 | 5.2 | 4,285 | 4.7 | 11884 | 4.8 |  | 3085 | 4.3 | 4417 | 4.8 | 3977 | 4.4 | 11479 | 4.5 |  |  |
| Skin and connective tissues | 2971 | 4.2 | 3891 | 4.4 | 3,480 | 3.9 | 10342 | 4.2 |  | 3643 | 5.0 | 5048 | 5.5 | 4378 | 4.8 | 13069 | 5.1 |  |  |
| ***Socio-economic status^1^*** |  |  |  |  |  |  |  |  |  |  |  |  |  |  |  |  |  |  |  |
| Lowest disadvantage | 19425 | 27.6 | 31300 | 35.4 | 23871 | 26.4 | 74596 | 29.9 | p<0.001 | 18657 | 25.8 | 31480 | 34.2 | 23260 | 25.5 | 73397 | 28.7 | p<0.001 | p<0.0001 |
| Low disadvantage | 11730 | 16.7 | 13420 | 15.2 | 17733 | 19.6 | 42883 | 17.2 |  | 12014 | 16.6 | 14311 | 15.6 | 18038 | 19.8 | 44363 | 17.4 |  |  |
| Moderate disadvantage | 14996 | 21.3 | 17561 | 19.9 | 16793 | 18.6 | 49350 | 19.8 |  | 15366 | 21.2 | 18319 | 19.9 | 16742 | 18.4 | 50427 | 19.7 |  |  |
| High disadvantage | 13615 | 19.3 | 17938 | 20.3 | 20164 | 22.3 | 51717 | 20.8 |  | 14277 | 19.7 | 18457 | 20.1 | 20128 | 22.1 | 52862 | 20.7 |  |  |
| Highest disadvantage | 7167 | 10.2 | 7586 | 8.6 | 11485 | 12.7 | 26238 | 10.5 |  | 7935 | 11.0 | 8503 | 9.3 | 12274 | 13.5 | 28712 | 11.2 |  |  |
| Unknown | 3480 | 5.0 | 528 | 0.6 | 338 | 0.4 | 4346 | 1.7 |  | 4193 | 5.8 | 873 | 1.0 | 678 | 0.7 | 5744 | 2.2 |  |  |
| ***Accessibility to services ^2^*** |  |  |  |  |  |  |  |  |  |  |  |  |  |  |  |  |  |  |  |
| Major cities | 49864 | 70.82 | 66607 | 75.4 | 70621 | 78.12 | 187092 | 75.1 | p<0.001 | 49205 | 67.9 | 66877 | 72.7 | 69228 | 76.0 | 185310 | 72.5 | p<0.001 | p<0.001 |
| Inner regional | 8572 | 12.17 | 8122 | 9.2 | 7629 | 8.4 | 24323 | 9.8 |  | 8920 | 12.3 | 8526 | 9.3 | 7890 | 8.7 | 25336 | 9.9 |  |  |
| Outer regional | 6990 | 9.93 | 7187 | 8.1 | 6667 | 7.4 | 20844 | 8.4 |  | 7779 | 10.7 | 8032 | 8.7 | 7208 | 7.9 | 23019 | 9.0 |  |  |
| Remote | 3472 | 4.9 | 4561 | 5.2 | 3253 | 3.6 | 11286 | 4.5 |  | 4441 | 6.1 | 5987 | 6.5 | 3874 | 4.3 | 14302 | 5.6 |  |  |
| Very remote | 1445 | 2.0 | 1668 | 1.9 | 1920 | 2.1 | 5033 | 2.0 |  | 1949 | 2.7 | 2114 | 2.3 | 2340 | 2.6 | 6403 | 2.5 |  |  |
| Unknown | 70 | 0.1 | 188 | 0.2 | 294 | 0.3 | 552 | 0.2 |  | 148 | 0.2 | 407 | 0.4 | 580 | 0.6 | 1135 | 0.4 |  |  |
| ***Comorbidities^3^*** |  |  |  |  |  |  |  |  |  |  |  |  |  |  |  |  |  |  |  |
| 0-1 | 19996 | 28.4 | 27128 | 30.7 | 28036 | 31.0 | 75160 | 30.2 | p<0.001 | 27112 | 37.4 | 36405 | 39.6 | 35501 | 39.0 | 99018 | 38.8 | p<0.001 | p<0.001 |
| 2-5 | 48123 | 68.3 | 58328 | 66.0 | 59142 | 65.4 | 165593 | 66.5 |  | 43211 | 59.7 | 52953 | 57.6 | 52381 | 57.5 | 148545 | 58.1 |  |  |
| 6+ | 2294 | 3.3 | 2877 | 3.3 | 3206 | 3.6 | 8377 | 3.4 |  | 2119 | 3.0 | 2585 | 2.8 | 3238 | 3.6 | 7942 | 3.1 |  |  |
| ***Outcome variables*** | |  |  |  |  |  |  |  |  |  |  |  |  |  |  |  |  |  |  |
| Individuals with at least one CT | 9065 | 12.9 | 11538 | 13.1 | 13259 | 14.7 | 33862 | 13.6 | p<0.001 | 9649 | 13.3 | 12371 | 13.46 | 13866 | 15.2 | 35886 | 14.0 | p<0.001 | p<0.001 |
| Individuals with 2+ CT | 2982 | 4.2 | 3981 | 4.5 | 5087 | 5.6 | 12050 | 4.8 | p<0.001 | 3685 | 5.1 | 5055 | 5.5 | 6055 | 6.7 | 14795 | 5.8 | p<0.001 | p<0.001 |
| Rate of any CT use (per 1000 index events) | 195.16 |  | 200.34 |  | 238.53 |  | 211.34 |  | p<0.001 | 223.21 |  | 228.55 |  | 277.25 |  | 243.00 |  | p<0.001 | p<0.001 |
| Rate of CT use (per 1,000 individuals with 2+ CT index events) | 2568.41 |  | 2547.10 |  | 2631.61 |  | 2588.05 |  | p=0.154 | 2769.61 |  | 2709.79 |  | 2882.25 |  | 2795.27 |  | p=0.001 | p<0.001 |
| Average individual cost (and SD) of CT scan per index event (in AUD)^4^ | 81.05 | 271.45 | 70.94 | 235.74 | 77.77 | 240.33 | 76.59 | 240.73 | p=0.010 | 88.68 | 302.44 | 77.34 | 256.1 | 86.23 | 268.39 | 84.08 | 275.64 | p=0.083 | p<0.001 |
| Total cost of CT scans during ascertainment period (sum, in millions AUD^4^) | 5.71 | n/a | 6.27 | n/a | 7.03 | n/a | 19.01 | n/a |  | 6.42 | n/a | 7.11 | n/a | 7.86 | n/a | 21.39 | n/a |  |  |
| Mean effective dose (and SD) in adults with at least one CT (mSv^5^) | 14.51 | 15.05 | 14.4 | 15.81 | 15.01 | 16.14 | 14.64 | 15.67 | p=0.019 | 12.67 | 14.5 | 12.47 | 14.26 | 13.35 | 16.03 | 12.83 | 18.26 | p<0.001 | p<0.001 |
| Cancer incidence for individuals with any CT (per 100000 individuals)^6^ | 85 | 104.6 | 83.1 | 109.5 | 84.4 | 109.6 | 84.2 | 107.9 | p=0.867 | 69.6 | 93 | 65.8 | 87.8 | 69.2 | 101.7 | 68.2 | 94.2 | p=0.905 | p<0.001 |
| Cancer mortality for individuals with any CT (per 100000 individuals)^6^ | 53 | 62 | 52.3 | 64.6 | 53.8 | 65.5 | 53.0 | 64.0 | p=0.873 | 40.8 | 51.3 | 39.1 | 49 | 41.6 | 56.4 | 40.5 | 52.2 | p=0.833 | p<0.001 |

*^1^ Socio-economic status measured by SEIFA-IRSD: Socio-Economic Indexes for Areas, Index of Relative Socio-Economic Disadvantage*

*^2^ Accessibility to services was measured using ARIA: Accessibility and Remoteness Index of Australia*

*^3^ Comorbidity: Number of Multipurpose Australian Comorbidity Scoring System conditions reported in hospitalisation data in the 5 years prior to the index date*

*^4^ AUD: Australian dollars adjusted to 2021 values using the Consumer Price Index.*

*^5^ Mean of effective dose accumulated within individuals across all scans during ascertainment period*

*^6^ LAR: Lifetime risk of cancer attributable to CT scanning undertaken during ascertainment period.*

*^7^ P-values report significance of changes from 2006 to 2015 within males and females separately*

*^8^ P-values report significances of differences between Males and Females in 2015*
